# Low-Velocity Impact Localization on a Honeycomb Sandwich Panel Using a Balanced Projective Dictionary Pair Learning Classifier

**DOI:** 10.3390/s21082602

**Published:** 2021-04-07

**Authors:** Zhaoyu Zheng, Jiyun Lu, Dakai Liang

**Affiliations:** 1 State Key Laboratory of Mechanics and Control of Mechanical Structures, Nanjing University of Aeronautics and Astronautics, Nanjing 210016, China; futusheng1989@gmail.com (Z.Z.); dkliang@nuaa.edu.cn (D.L.); 2 College of Civil Aviation, Nanjing University of Aeronautics and Astronautics, Nanjing 210016, China

**Keywords:** impact localization, projective dictionary pair learning, fiber Bragg grating, structural sparse representation

## Abstract

Carbon-fiber aluminum honeycomb sandwich panels are vulnerable to low-velocity impacts, which can cause structural damage and failures that reduce the bearing performance and reliability of the structure. Therefore, a method for locating such impacts through a sensor network is very important for structural health monitoring. Unlike composite laminates, the stress wave generated by an impact is damped rapidly in a sandwich panel, meaning that the signal qualities measured by different sensors vary greatly, thereby making it difficult to locate the impact. This paper presents a method for locating impacts on carbon-fiber aluminum honeycomb sandwich panels utilizing fiber Bragg grating sensors. This method is based on a projective dictionary pair learning algorithm and uses structural sparse representation for impact localization. The measurement area is divided into several sub-areas, and a corresponding dictionary is trained separately for each sub-area. For each dictionary, the sensors are grouped into main sensors within the sub-area and auxiliary sensors outside the sub-area. A balancing weight factor is added to optimize the proportion of the two types of sensor in the recognition model, and the algorithm for determining the balancing weight factor is designed to suppress the negative effects on the positioning of the sensors with poor signal quality. The experimental results show that on a 300 mm × 300 mm × 15 mm sandwich panel, the impact positioning accuracy of this method is 96.7% and the average positioning error is 0.85 mm, which are both sufficient for structural health monitoring.

## 1. Introduction

The honeycomb structure is a structure from nature, which has inspired research in many fields. A graphene and carbon nanotube structure can provide high-rate capability and stretchability of a battery and electrodes [1,2]. A honeycomb structure can also be introduced to the surface engineering of polymeric membranes; this structure can provide high solvent permeance and a stable performance [3]. In aerospace structures, honeycomb panels are widely used as wall panels and wing panels. Carbon-fiber aluminum honeycomb sandwich panels are lightweight, have strong load-bearing performance, and provide vibration isolation and energy absorption [4,5]. However, when subjected to low-velocity impacts, the honeycomb panel structures are prone to damage such as core collapse and surface layer tearing, which affects the performance and reliability of the material structure [6,7]. Under visual observation, the damage caused by low-velocity impacts is usually not obvious, which makes damage detection difficult [8]. However, by arranging a sensor network on the structure to measure the response signal caused by an impact, the impact can be localized via a positioning algorithm, which is very important for structural health monitoring [9,10]. Fiber Bragg grating (FBG) sensors can be conveniently arranged on various material structures, have the advantages of anti-electromagnetic interference and integrated sensing and communication functions, and are very suitable for constructing a sensor network for impact positioning [11].

Researchers often use machine learning algorithms for impact positioning [12]. The measurement area is divided as many grids, and impacts are applied at the grid centers or intersections to obtain response signals at different positions. These signals are then processed as training samples for machine learning algorithms. When positioned, the unknown impact response signal is classified or predicted via the recognition algorithm. Pratik Shrestha and Yurim Park et al. developed an error outlier with weighted median absolute deviation threshold algorithm for localizing the impact on complex composite wing structures embedded with three-multiplexed FBG sensors [13]. A high-speed interrogator with a sampling frequency of 100 KHz was used to obtain impact response signals and to guarantee the measurement accuracy. Gang Zhao and Shuxin Li et al. extracted the amplitude of the third-order natural frequency of impact response signals and then established a third-order vector matrix model and used a third-order sum of squares of deviations to predict positions [14]. During the test, four FBG sensors were placed along the 45° angle on the surface of the carbon-fiber reinforced polymer (CFRP) and their signals can be monitored by interrogator SM130. Mingshun Jiang et al. used an FBG interrogation system with a sampling frequency of 1 kHz and extreme learning machine technology algorithm to locate the impact position [15]. Jiyun Lu and Bangfeng Wang et al. extracted feature vectors from FBG signals by means of a wavelet packet decomposition algorithm and adopted support-vector regression to realize impact locations [12]. Shizeng Lu et al. set an FBG interrogator system which was composed of four photo-electric detectors and a high-speed data acquisition with a sampling frequency of 10 MHz in order to capture wavelength shifts from four FBG sensors on CFRP [16]. Least squares support vector machines (LS-SVM) model training was utilized to predict the impact position, which achieved localization errors of less than 5 mm. All works discussed above were about the impacts on composite laminate structures.

The positioning method based on machine learning has good applicability to different material structures and different shapes of targets, and it has achieved good results in the impact positioning problem for composite material laminates. However, the characteristics of vibration and energy absorption of honeycomb sandwich panels cause more difficulties for low-velocity impact positioning of this type of material structure, and impact-positioning research for sandwich panels is still in its infancy. The stress wave generated by an impact in a honeycomb sandwich structure is attenuated much more rapidly than that in a laminate, which has two consequences [17]. First, the signal qualities measured by different sensors vary greatly. The time–domain waveform measured by a sensor far from the impact position is quickly submerged by noise, and effective features cannot be extracted. The signal measured by this type of sensor will have a negative impact on the positioning algorithm, whether it is used for training or testing. Second, the impact response signal of a single impact loading cannot contain all the features required for the positioning algorithm.

Aiming at the two aforementioned shortcomings, this paper proposes a positioning method based on structural sparse representation with a projective dictionary pair learning algorithm (hereinafter referred to simply as DPL) [18]. DPL is a discriminative dictionary learning (DL) algorithm, in which the training phase and the classification phase are relatively independent. In the process of DL, the internal data structure of the sample is stable and clear. This character makes the corresponding parts of different sensors in the learned dictionary easy to be determined. In the proposed method, the proportion of different sensors could be adjusted only by the modification of the discriminant in the classification phase. By simultaneously learning a synthesis dictionary and an analysis dictionary, DPL can improve the performance of dictionary identification and expression. In common DL algorithms, the calculations of the minimum L_0_ norm or the minimum L_1_ norm are computationally complex. By constructing an analysis dictionary, DPL avoids the above calculations in the training and classification process. Every step in the process has a closed solution, which makes DPL much faster than other DL algorithms. As a discriminative DL algorithm, DPL can take into account the classification ability and operation speed, which is suitable for impact localization. Aiming at the problem of variable signal qualities among sensors, the proposed method divides and combines the measurement areas and trains a dictionary separately for each sub-area. The sensors are grouped into the main sensors inside the sub-area and the auxiliary sensors outside of the sub-area. The proportion of the two types of sensors in the positioning algorithm are optimized by adding a balancing weight factor (BWF) to each sub-classifier, by which the interference of the sensors with poor signal quality to the positioning method is suppressed. The experimental results show that the method proposed herein is good for low-velocity impact positioning for sandwich panels and the positioning accuracy can be improved by increasing the number of training samples.

## 2. FBG Sensing Technique

An FBG measures the impact response signal by measuring the shift of the center wavelength of the reflection spectrum. Affected by strain and temperature, the center wavelength of the FBG is expressed as follows [12]:

(1) $$\frac{\Delta \lambda_{B}}{\lambda_{B}}=\left\{1-\left(\frac{n^{2}}{2}\right)\left[P_{12}-\nu \left(P_{11}+P_{12}\right)\right]\right\}\varepsilon +\left[\alpha +\frac{\left(dn/dT\right)}{n}\right]\Delta T,$$

where $\lambda_{B}$ is the Bragg wavelength of the FBG, n is the average refractive index of the fiber, $P_{ij}$ are the Pockel (piezo) coefficients of the stress-optic tensor, v is Poisson’s ratio of fiber, α is the thermal expansion coefficient of the fiber, ε is the strain on the sensor, and ΔT is the change in temperature. In this experiment, a temperature-compensation sensor was used to eliminate the influence of temperature changes on the measurement. When only the influence of the strain on the sensor is considered, Equation (1) simplifies as follows:

(2) $$\frac{\Delta \lambda_{B}}{\lambda_{B}}=P_{e}\varepsilon ,$$

where $P_{e}$ is the elasto-optic coefficient. As can be seen, $\Delta \lambda_{B}$ is related linearly to the strain, so the stress wave generated by an impact can be measured by measuring the change of the center wavelength of the sensor.

## 3. Experimental Setup and Specimen Analysis

**Figure 1:**
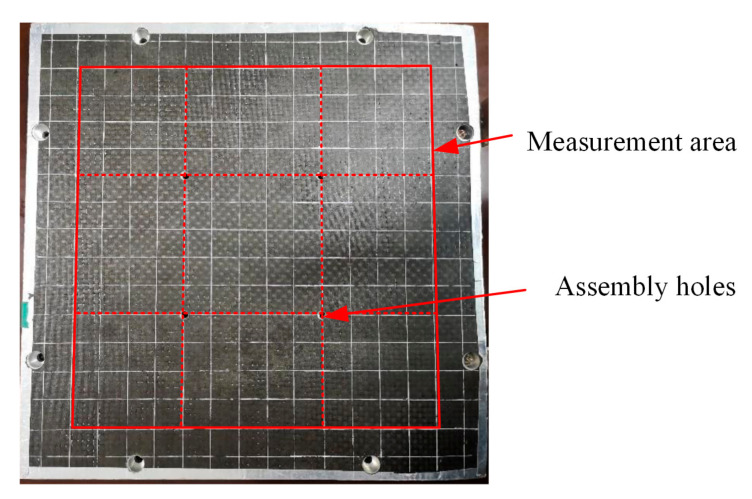
Honeycomb sandwich specimen and measurement area.

The test specimen in the experiment was a 300 mm × 300 mm × 15 mm carbon-fiber aluminum honeycomb sandwich panel with assembly holes which was produced as a wall or wing panel for a microsatellite. The upper and lower surface layers of the sandwich panel were made of T700/AG80 carbon-fiber plies with a thickness of 1 mm. The honeycomb core was a hexagonal aluminum honeycomb core with a wall thickness of 0.3 mm. A photograph of the specimen is shown in Figure 1.

Avoiding the edge cladding and edge assembly holes, the actual measurement area was the red frame shown in Figure 1. The measurement area was gridded into squares with a side length of 18 mm, and impacts were applied at the 196 grid intersections. There were four assembly holes near the center, which naturally divided the measurement area into nine square areas. The stress wave propagation on the bottom layer of the honeycomb panel was suppressed by the assembly holes. Considering the entire measurement area would be monitored, all nine square areas in Figure 1 needed to be deployed with FBG sensors. Obviously, when the sensors are pasted near the center of the nine square areas, the best distance sensitivities can be obtained. Therefore, nine FBG sensors were pasted on the back of the sandwich panel, and a temperature-compensation FBG was pasted onto another test piece of the same specification.

Taking the geometric center of the measurement area as the origin, the horizontal and vertical center lines of the specimen are the X and Y axes to establish a coordinate system. The numbers and pasting positions of the nine sensors are given in Table 1.

**Figure 2:**
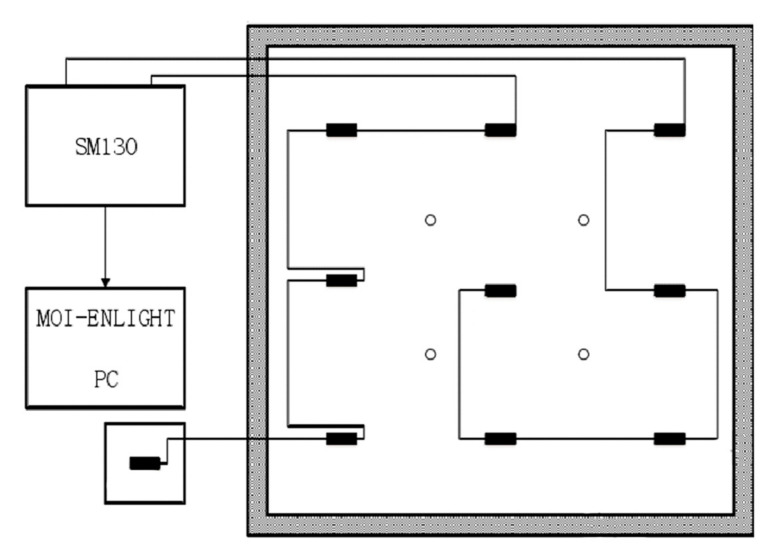
Diagram of the measurement system and the sensor layout.

**Figure 3:**
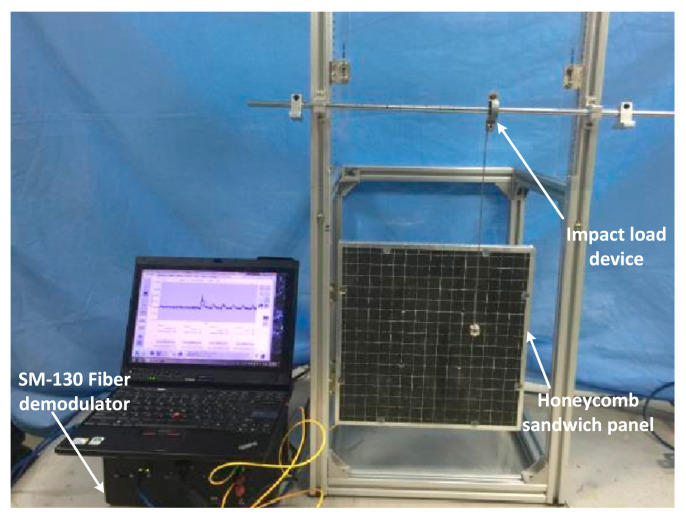
Photo of the measurement system and loading frame.

The test piece was fixed on the test frame, and impacts were generated by a 10-g oblique pendulum. The metal connecting rod had a pendulum length of 350 mm, and the release angle of the oblique pendulum was 45°. An SM130 demodulator was used to record the wavelength change of the sensors, and the sampling frequency was 1 kHz. In this study, a workstation with an E5-2670 CPU and a frequency of 3.3 GHz was deployed as the computer environment. A diagram and a photograph of the experimental system are shown in Figure 2 and Figure 3, respectively.

**Figure 4:**
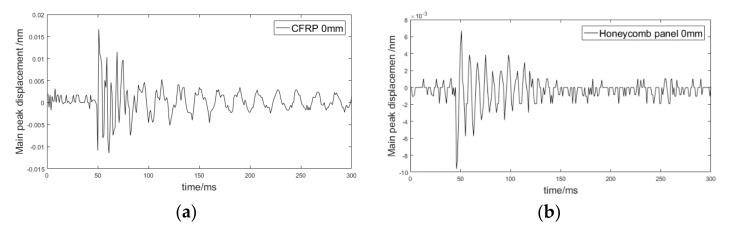

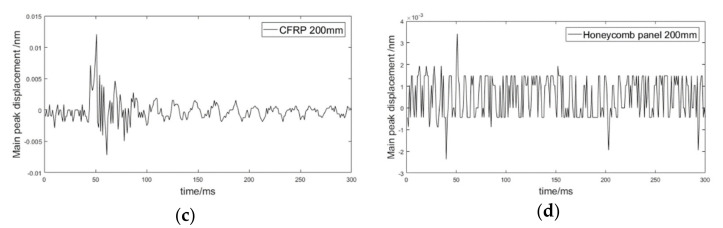
Time-domain response signals of the sandwich panel and carbon-fiber reinforced polymer (CFRP) laminate: (**a**) CFRP with sensor 0 mm from impact position; (**b**) honeycomb panel with sensor 0 mm from impact position; (**c**) CFRP with sensor 200 mm from impact position; (**d**) honeycomb panel with sensor 200 mm from impact position.

To illustrate further the impact response characteristics of the sandwich panel, a 300 mm × 300 mm × 2.1 mm CFRP laminate was used as the control specimen. The two specimens were loaded with the same impacts, which were from FBG1 0 mm and 200 mm respectively, and the recorded time–domain signals are shown in Figure 4.

**Figure 5:**
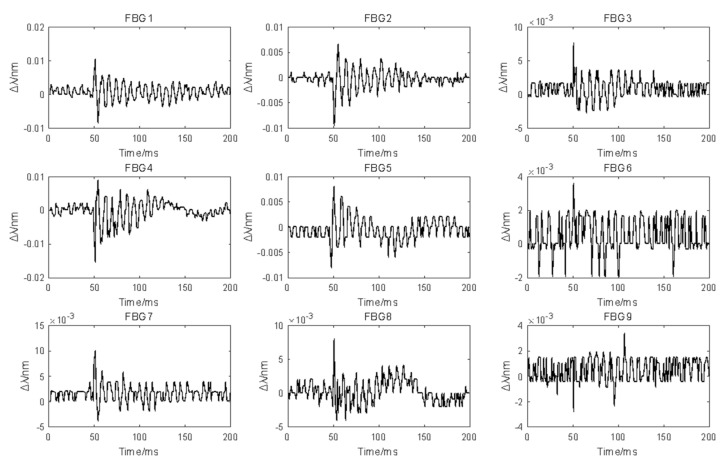
Time-domain response signals of the impact (−81 mm, 81 mm) measured by the nine FBG sensors.

Figure 4 shows that compared with the CFRP laminate, the amplitude of the response signal for the sandwich panel was smaller and the attenuation was faster. Comparing Figure 4a,b, the effective time–domain part for the sandwich panel was significantly shorter than that for the CFRP panel. When the sensor was 200 mm away from the impact position, as shown in Figure 4d, the measured signal was almost unusable. When the impact was applied at the intersection (−81 mm, 81 mm), which was the intersection point nearest to FBG1, the time–domain response signals measured by the nine sensors were as shown in Figure 5.

**Figure 6:**
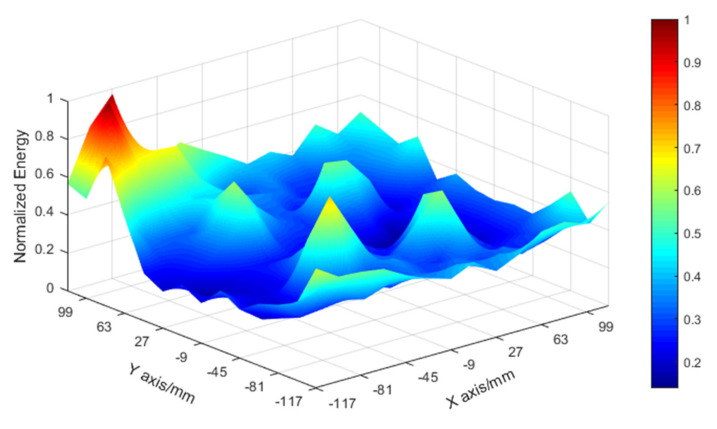
Normalized energy contour map of a sandwich panel sensed by FBG1.

Figure 5 shows that FBG1, FBG2, FBG4, and FBG5 measured clearer time–domain waveforms, whereas FBG6, FBG8, and FBG9, which were far from the impact position, had almost no clear waveforms in their measured response signals. Signals such as the latter, whether used for training or testing, may have a negative effect on the positioning algorithm. Next, each grid intersection in the measurement area was impact loaded, the signal energy measured by FBG1 was calculated and normalized, and the resulting energy contour map is shown in Figure 6. As can be seen, the energy of the response signal decreased with increasing distance between the sensor and the impact. Obviously, higher signal energy means better signal quality.

**Figure 7:**
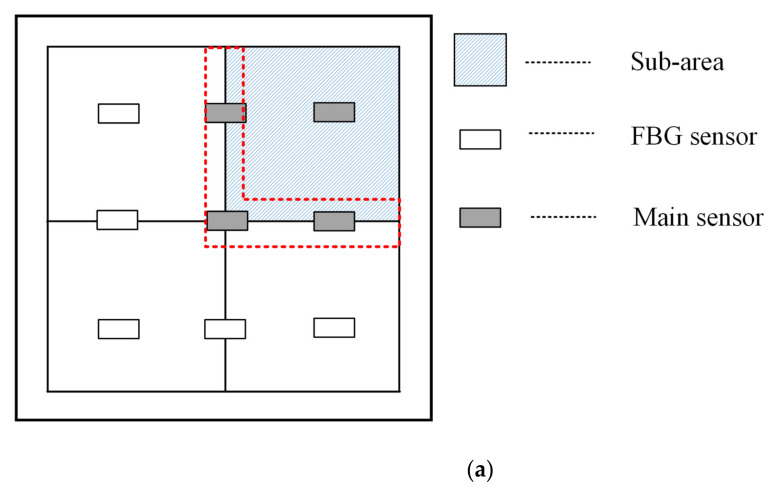

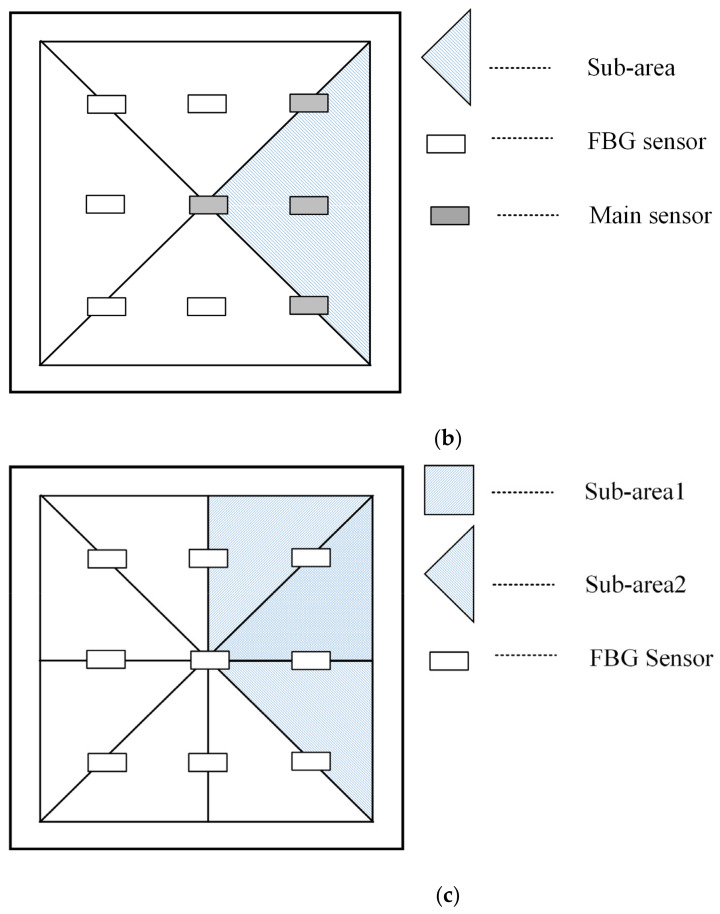
Division and sub-areas of the measurement area: (**a**) square mode; (**b**) triangle mode; (**c**) division mode in the proposed method.

Figure 5 and Figure 6 show that in the array of nine sensors, the response signals measured by those closer to the impact position had better signal quality, whereas the signal quality measured by those far from the impact position was poor. In general classification methods, signals of different qualities are usually not processed in a targeted manner. Additionally, in our research, there were two optional division modes, as shown in Figure 7a,b and under sensor layout in Figure 2. Herein, the measurement area is segmented and combined to achieve the optimal sensor configuration. The segmentation of the measurement area is shown in Figure 7.

As shown in Figure 7a, FBG2, FBG3, FBG5, and FBG6 were selected as the main sensors, which were given a higher weight in classification. In Figure 7b, FBG3, FBG5, FBG6, and FBG9 were selected as the main sensors. In the actual experiment process, when the impact loaded close to the boundary of the sub-area, as shown in the red dashed box in Figure 7a, the sub-area is easy to be judged incorrectly. Improper main sensors and a sub-classifier were selected, which caused massive location errors. This situation can be avoided by combining the division modes in Figure 7a,b, which was shown in Figure 7c. The measurement area was divided into eight triangular sub-blocks. Two adjacent triangular sub-blocks were assembled into a measurement sub-area (sub-areas 1 and 2 are shown in Figure 7a), giving a total of eight sub-areas. This division and combination form has overlap and redundancy, but it is effective at preventing the impact signal from falling at the junction of the two sub-areas. The four FBGs in each sub-area are the main sensors, and the five FBGs outside the sub-area are the auxiliary sensors. To position the impact signal, the sub-area where the impact was located is first determined by simply comparing the maximum value of the total energy of the main sensor group in each region. The sensor grouping of each sub-area is given in Table 2.

## 4. Sparse Representation Classifier Based on Balanced-DPL

### 4.1. DPL Algorithm

Sparse representation compresses and classifies the signal by combining it linearly with a few atoms in the dictionary [19]. In sparse representation, a proper DL algorithm improves the representation performance and discrimination performance of a complete dictionary [20,21]. As the present study is concerned with signal classification, more concern is given to the discrimination performance when choosing the DL algorithm. Compared with K-means SVD (K-SVD), label consistent K-SVD, discriminative K-SVD, and other holistic DL algorithms, DPL trains the sub-dictionaries separately for each category during the DL process, so it pays more attention to the dictionary identification ability during the learning process [22,23]. Compared with the Fisher discrimination DL algorithm (FDDL), DPL has a closed solution for every step of the dictionary learning and solving and so is faster [24].

A typical DL model is as follows:

(3) $$J_{\left(D,X\right)}=\arg\underset{\left(D,X\right)}{\min}\left\{||Y-DX|{|}_{F}^{2}+\lambda ||X|{|}_{p}+f\left(X,D\right)\right\},$$

where Y is the training sample, D is the sparse dictionary, and X is the coding coefficient matrix. The first term in Equation (3) guarantees the expression performance of the dictionary, the second term guarantees the sparsity of the coding matrix, and the third term is the objective function related to improving the discrimination performance.

In DPL, not only is dictionary *D* learned, but also an analysis dictionary $P\in R^{mk\times p}$, X=PY is learned, where K is the number of categories and m is the number of atoms of each category. The learning model is as follows:

(4) $$\left\{P^{*},D^{*}\right\}=\arg\underset{P,D}{\min}\left\{||Y-DPY|{|}_{F}^{2}\;+\Psi \left(D,P,X\right)\right\},$$

where $\Psi \left(D,P,X\right)$ is the constraint item related to the discriminative ability. The dictionary pair is built from the analysis dictionary $P=\left[P_{1};\cdots ;P_{i};\cdots ;P_{k}\right]$ and the synthesis dictionary, where $D_{j}$ and $P_{j}$ are the sub-dictionaries of each category. The purpose of P is to project Y, and that of D is to reconstruct Y. When the signal satisfies certain incoherence conditions, the sample can be represented by only the sub-dictionary of its category. To improve the discrimination of the dictionary, when $P_{j}$ and the sample are not in the same category, then the projection of the sample by the sub-dictionary should be in a nearly null space:

(5) $$P_{k}Y_{i}\approx 0,\forall k\neq i.$$

Because X=PY, the constraints on the coding coefficient matrix X can be obtained through those of $P_{k}X_{k}$ The objective function of DPL becomes as follows:

(6) $$\left\{P^{*},D^{*}\right\}=\arg\underset{P,D}{\min}\sum_{i=1}^{k}||Y_{i}-D_{i}P_{i}Y_{i}|{|}_{F}^{2}\;+\gamma ||P_{i}{\bar{Y}}_{i}|{|}_{F}^{2},s.t.||d|{|}_{2}^{2}\leq 1,$$

where the scalar γ adjusts the weight of the two constraints, and $\;{\bar{Y}}_{i}$ is the sample not including those from category  i. Equation (6) is non-convex and can be changed into a convenient solution form by adding a relaxation term. In actual calculations, the objective function of DPL DL is as follows:

(7) $$\left\{P^{*},X^{*},D^{*}\right\}=\arg\underset{P,A,D}{\min}\sum_{i=1}^{k}||Y_{i}-D_{i}P_{i}Y_{i}|{|}_{F}^{2}\;+\tau ||P_{i}Y_{i}-X_{i}|{|}_{F}^{2}+\gamma ||P_{i}{\bar{Y}}_{i}|{|}_{F}^{2},s.t.||d|{|}_{2}^{2}\leq 1,$$

where τ is a scalar constant.

### 4.2. Balanced-DPL Classifier

During the DPL learning process, the learning of $P_{j}^{*}$ increases the values of the coding coefficients of samples not in class j and decreases those of class *j*. The sparse representation of the sample is $D_{j}^{*}P_{j}^{*}Y_{j}$, which means that $||Y_{k}-D_{k}^{*}P_{k}^{*}Y_{k}|{|}_{F}^{2}$ has a relatively small value. Meanwhile,$\;P_{j}^{*}Y_{i},i\neq j$ is small and $||Y_{i}-D_{j}^{*}P_{j}^{*}Y_{i}|{|}_{F}^{2}$ is greater than $||Y_{j}-D_{j}^{*}P_{j}^{*}Y_{j}|{|}_{F}^{2}$. So when testing, if the test sample belongs to class j, then the reconstruction residual of the sub-dictionary of class j, i.e.,$\;||y-D_{j}^{*}P_{j}^{*}y|{|}_{2}^{2}$ is smaller than $||y-D_{i}^{*}P_{i}^{*}y|{|}_{2}^{2}$. Therefore, the solution discriminant of the test sample in DPL is as follows:

(8) $$identity\left(y\right)=\underset{i}{\mathrm{argmin}}||y-D_{i}P_{i}y|{|}_{2}.$$

As can be seen, the classification of DPL relies on the reconstruction residuals of the test samples from the sub-dictionaries, and low-quality signals will affect the classification result. In Table 3, the sensors are divided into the main sensor group and the auxiliary sensor group in each sub-area, and obviously the quality of the auxiliary sensor group is poor. A BWF $w_{B},\;0\leq w_{B}\leq 1$ is added to reduce the proportion of auxiliary sensors in the classifier, and the balancing weight vector is defined as follows:

(9) $$W_{B}=\left[\underset{\overset{⏟}{K_{m}}}{1,\cdots ,1},\underset{\overset{⏟}{K_{a}}}{w_{B},\cdots ,w_{B}}\right]\in R^{\left(K_{m}+K_{a}\right)}\;,$$

where the sample parts corresponding to the main sensors are assigned the value 1, and those corresponding to the auxiliary sensors are assigned the value $w_{B}$. Equation (9) then becomes as follows:

(10) $$identity\left(y\right)=\underset{i}{\mathrm{argmin}}||yW_{B}-D_{i}P_{i}yW_{B}|{|}_{2}.$$

**Figure 8:**
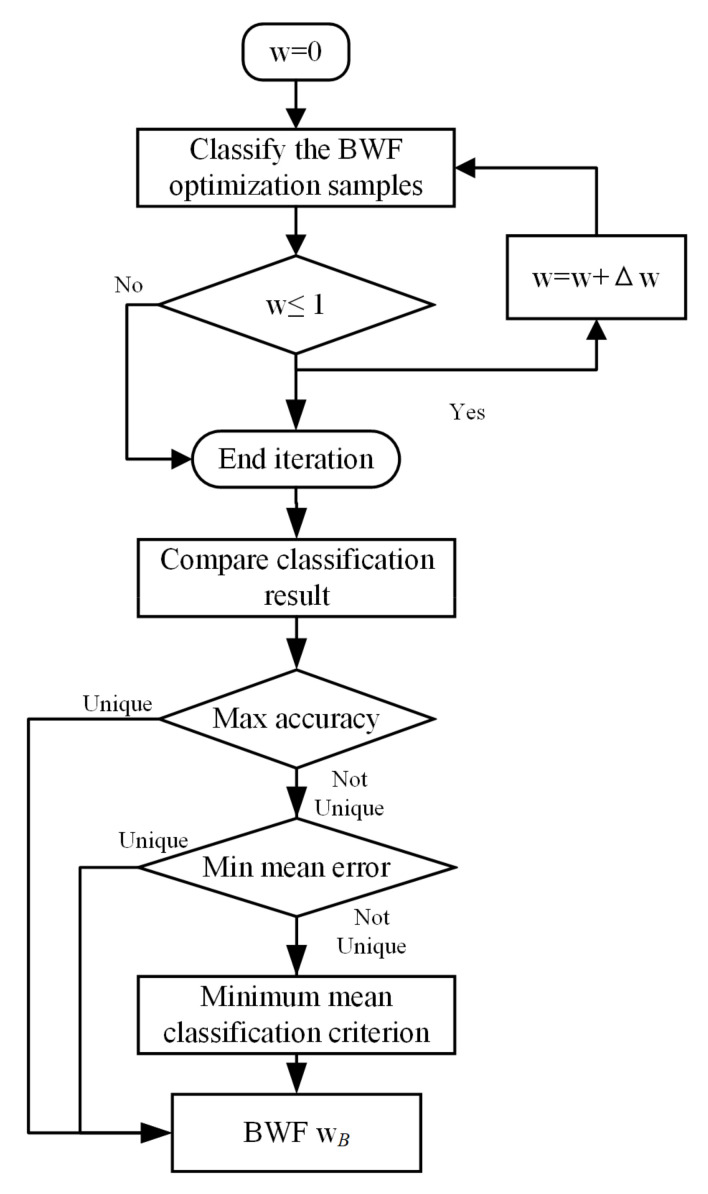
Solution process for balancing weight factor (BWF).

Equation (10) is the classifying solution of balanced-DPL (B-DPL), for which it is important to choose the correct value of $w_{B}$. For different sub-areas, there are different main and auxiliary sensors, and the corresponding optimal BWF is also different. Three indicators are used to evaluate the effectiveness of a positioning algorithm: (i) positioning accuracy, (ii) mean positioning error, and (iii) classification criteria $||y’-D_{i}P_{i}y’|{|}_{2}$. By positioning the samples and comparing these three indicators in turn, the optimal BWF is obtained. The algorithm flow is shown in Figure 8.

In the solution process, the BWF starts from zero and is increased to unity with a step length, and the positioning result of every step is compared. Note that the maximum accuracy and minimum mean error may correspond to several different values of $w_{B}$. If the $w_{B}$ obtained by the maximum accuracy is not unique, then the mean errors are compared, and if the $w_{B}$ is still not unique, then the classification criteria are compared.

### 4.3. Method Processing

**Figure 9:**
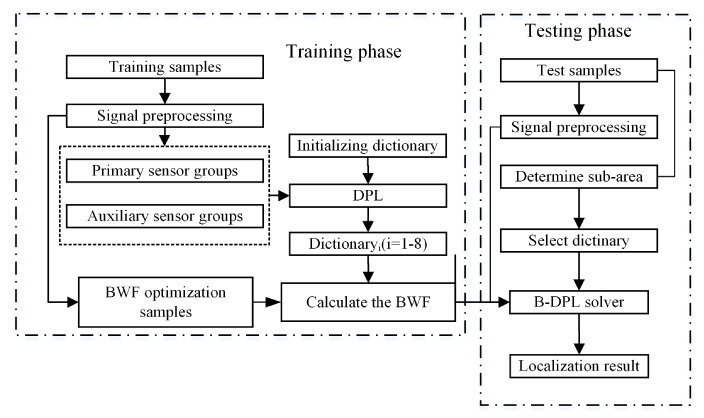
Flow chart of the localization method.

The proposed positioning method has two phases, i.e., training and testing. A flow chart of the entire positioning method is shown in Figure 9.

In the training phase, the measurement area is divided into eight sub-areas, each of which has four main FBG sensors and five auxiliary sensors. The impact response signals are processed through signal preprocessing and feature extraction to generate training samples for the corresponding sub-areas. Here, each dictionary of a sub-area contains the samples of all the intersections on the specimen to avoid the situation in which there is no corresponding category in the dictionary when the sub-area is judged incorrectly. Each sub-area acquires a trained dictionary from DPL, and the BWF is calculated by the algorithm in Figure 8. The samples used to optimize the BWF could be a set of independent samples or from training samples in cross-validation. When the impact is prelocalized, there is a high probability of the sub-area being selected correctly; therefore the samples for calculating the BWF only need to be those in the corresponding sub-areas. In the testing phase, the test samples were obtained in the same way as in the training phase, then sub-areas were determined by the signal energy. Finally, the corresponding balanced-DPL classifier and BWF were called to solve the positioning.

### 4.4. Signal Preprocessing and Feature Extraction

**Figure 10:**
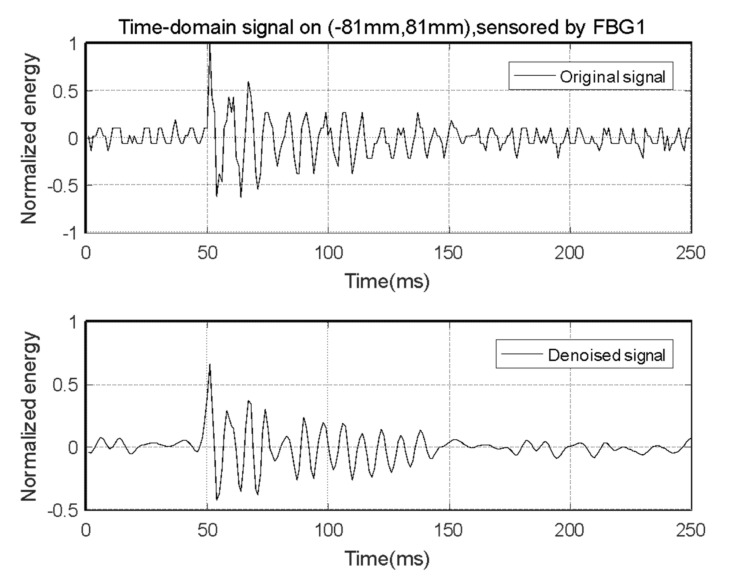
Wavelet noise-reduction effect of time-domain impact response signal.

The response signal measured by an FBG sensor is processed by a wavelet transform denoise technique with the sqtwolog-threshold method using three-layer Daubechies-8 (DB8) wavelet decomposition as the noise reduction algorithm [25]. The noise–reduction effect is shown in Figure 10.

As can be seen, the signal after wavelet noise reduction is better at retaining the impact response waveform after denoising. The average mean square error of the signal sensed by the main sensors before and after noise reduction was 0.101.

**Figure 11:**
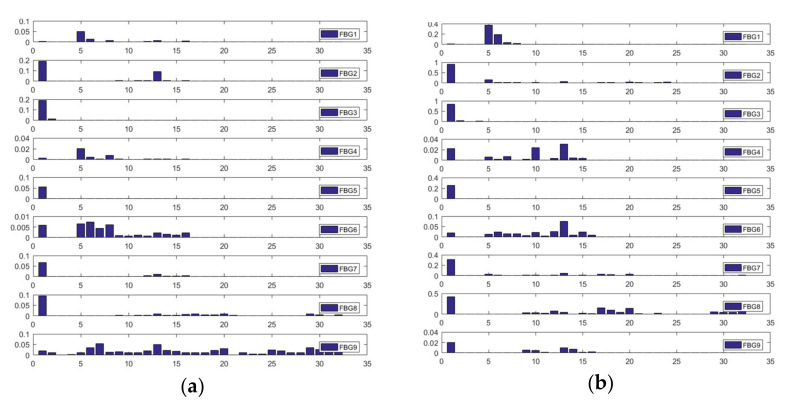

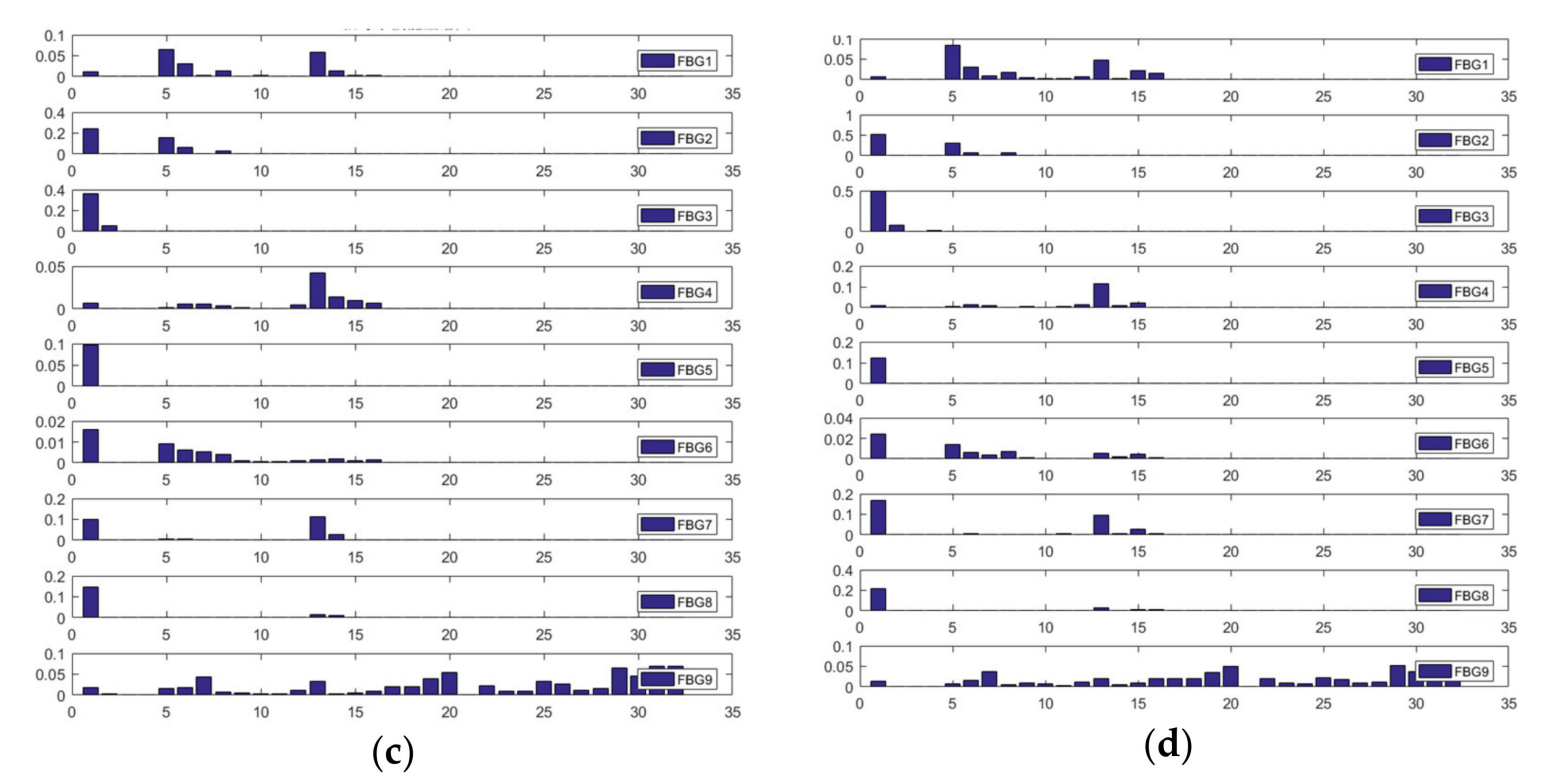
Wavelet packet energy spectrum of repeated impacts at (−81 mm, 81 mm): (**a**) first impact; (**b**) second impact; (**c**) third impact; (**d**) fourth impact.

The features were gained by wavelet decomposition and principal component analysis (PCA). The signal 256 ms before and after the stress wave arrives was selected for wavelet decomposition. The five-layer DB8 wavelet decomposition of response signals at the impact position (−81 mm, 81 mm) are shown in Figure 11.

Figure 11 shows that although the impact position was the same, the decomposition coefficients of each level were not. For the positioning problem, it was necessary to extract the components related to the impact position. With the impact position as the category label, the Fisher discrimination criterion for each level of decomposition coefficient was calculated, respectively [26]. According to the calculation results, orders 1, 5–7, 10, and 12–16 had higher Fisher discrimination criteria, and these orders were selected as the characteristics of the samples.

The frequency domain part of the signal were feature extracted by PCA, and the top 90% covered features were selected [27,28]. The length of PCA features in each sub-area is shown in Table 3. In order to ensure that the feature length of each sub-region is consistent, the length of the main sensors’ PCA feature is selected as 45, and the length of the auxiliary sensors’ PCA feature is 40 in this research.

## 5. Experiment and Results

### 5.1. Sample Collection and Selection

In the experiment, each grid intersection of the measurement area was impacted seven times. The signals that were measured the first six times were used for DL, and there were 1176 training samples divided into 196 categories. The seventh time, 80 signals scattered in the measurement area were selected as the optimized samples for the BWF, and 20 samples were allocated in each sub-area. Of the remaining samples, 30 were selected as test samples.

### 5.2. Parameter Selection

DPL involves three parameters: *λ*, *τ*, and *m*. In this method, the number of dictionary atoms is compressed, so *m* is the number of times that training samples are collected. In the experiment group, we had m=6. Then *λ* and *τ* were obtained by six-fold cross-validation. In the solution of the BWF, the iteration step size Δw was 0.05, and the calculated BWF for each sub-area is given in Table 4.

### 5.3. Control-Group Settings

There are three control groups (CGs) in this experiment.

1. CG1 involved DPL with BWF=1. In this group, the positioning algorithm model degenerated to the original DPL. The measurement area was not divided, and all the sensors had the same weight in the classifier.
2. CG2 involved DPL with BWF=0. In this group, the measurements were divided in the same way as in the experiment group, but when the sub-area of impact was determined, only the main sensors were selected to obtain the impact response signal.
3. CG3 involved the FDDL algorithm. In this group, the measurements were divided in the same way as in the experiment group. The global mode classifier was chosen, and the FDDL algorithm was optimized with BWF (hereinafter referred to simply as B-FDDL). The classification solution was as follows:
  (11) $$\hat{x}=\underset{x}{argmin}\left\{||y-DW_{B}’x|{|}_{2}^{2}+\gamma ||x|{|}_{1}\right\},$$
  (12) $$identity\left(y\right)=\arg\underset{i}{min}||yW_{B}’-D_{i}W_{B}’{\hat{x}}_{i}|{|}_{2}^{2}\;+\omega ||\hat{x}-m_{i}|{|}_{2}^{2}.$$
4. CG4 involved the SVM algorithm, which was used in the research of Lu et al. [12]. The layout of the sensors, grid setting, and feature exaction of GC3 were the same as this method. In GC3 the measurement area was not divided.
5. CG5 involved the ELM algorithm, which was used in the research of Jiang et al. [15]. The layout of the sensors, grid setting, and feature exaction of GC3 were the same as this method. In GC3 the measurement area was not divided.

All the parameters in the CGs were obtained by six-fold cross-validation, and if the BWF was needed (GC3), the calculations of the BWF were designed according to Figure 8.

### 5.4. Results and Discussion

**Figure 12:**
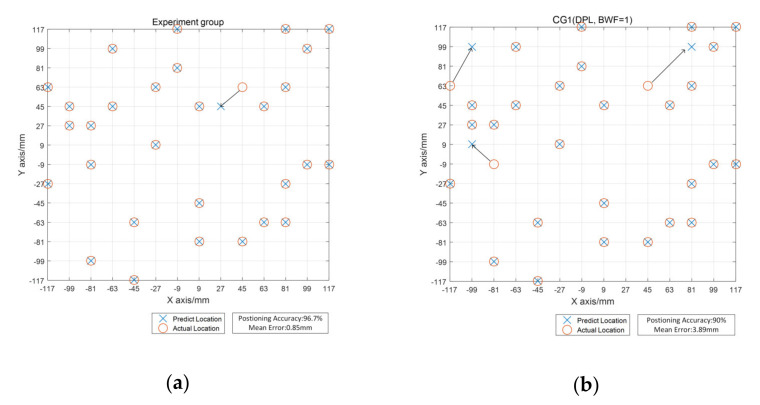

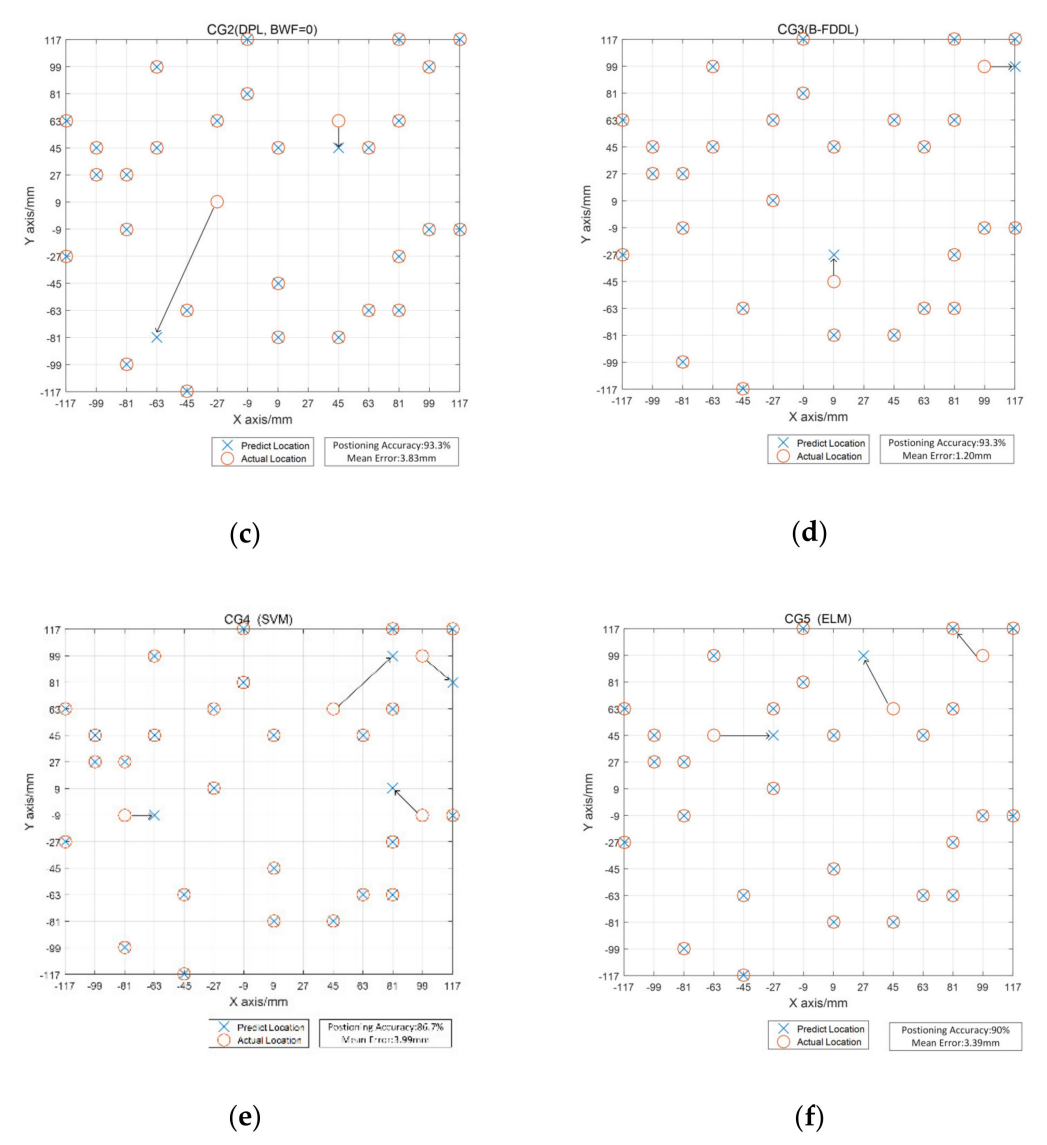
Impact localization results for (**a**) experiment group (B-DPL); (**b**) control group 1 (CG1, DPL, BWF = 1); (**c**) CG2 (DPL, BWF = 0); (**d**) CG3 (B-FDDL); (**e**) CG4 (SVM); (**f**) CG5 (ELM).

The positioning result of 30 test samples was obtained by the proposed method and the five control methods, and the results are shown in Figure 12.

Figure 12 shows that the experiment group gave the best positioning result, with a positioning accuracy of 96.7% and an average error of 0.85 mm, which were better than those of other CGs. In CG1 all nine FBG sensors were treated equally and in CG2 only four main sensors were used in classification when the sub-area was determined. Compared with CG1, CG2 had better positioning accuracy and a smaller mean error. However, when the impact was load at (9 mm, −27 mm), a massive positioning error occurred. By backtracking the algorithm flow, the cause of this error was discovered. Sub-area 6 is the proper sub-area for the test sample (9 mm, −27 mm), but this sample was incorrectly judged to be Sub-area 5, which caused the improper main sensor to be selected. It should be mentioned that in the experimental group and CG1, the sample was also judged to be Sub-area 5. However, in CG2 only the main sensors were used, which led to the absence of sensors with better quality and a massive error. Of the five CGs, CG3 involving the B-FDDL algorithm gave the best positioning result, with a positioning accuracy of 93.3% and an average positioning error of 1.20 mm. CG4 (SVM) and CG5 (ELM) did not show competitiveness in the experiment, even compared with CG2 in which the original DPL was selected.

**Figure 13:**
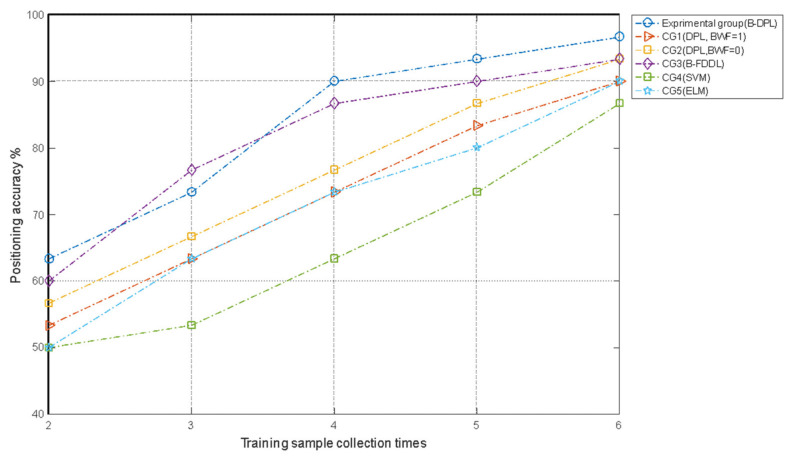
Positioning accuracy with different numbers of collections of training samples.

For the recognition method of sparse representation and other machine learning algorithms, the discriminative ability is related to the number of atoms (training samples) in the same category. It is feasible to improve the performance of the positioning method by increasing the number of training samples. Figure 13 shows the accuracy of the positioning algorithm when two, three, four, and five impact loadings were used for the training samples.

Figure 13 shows that when collecting training samples more often, the positioning methods become more accurate. The positioning accuracies of the B-DPL and B-FDDL algorithms are higher than those of the other four control methods. At four atoms, the improvement in the positioning accuracies of the B-DPL and B-FDDL algorithms begin to slow down, indicating that these two methods have lower requirements for the number of training samples. The positioning accuracy of DPL (BWF = 0) is higher than that of DPL (BWF = 1), indicating that when the number of training samples is small, the measurement area can be divided into sub-areas to improve the positioning accuracy. The result of CG5 (ELM) is comparable to that of CG1 (original DPL) but the result of CG5 is obviously inferior to the experimental group (B-DPL) and CG3 (B-FDDL).

Figure 13 also shows that as the CG, B-FDDL gave an equivalent positioning accuracy to that of the experiment group. However, considering the computational speed, the proposed B-DPL positioning algorithm has obvious advantages. In the training phase, B-DPL took 197.349 s while B-FDDL took 132931.441 s. To locate 30 test samples in the testing phase, B-DPL took 0.029 s while B-FDDL took 281.232 s. Considering the requirement of calculation speed in impact localization, the algorithm proposed is a better choice.

## 6. Conclusions

This paper has presented a low-velocity impact positioning method for honeycomb sandwich panels. This method uses (i) an FBG sensor network to record the stress-wave signal generated by an impact and (ii) a BWF to optimize the DPL algorithm to perform sparse representation classification to localize the impact signal. Aiming at the problem of rapid attenuation of the stress wave in the sandwich panel, the measurement area is divided into multiple sub-areas, and the sensor network is divided into main and auxiliary sensors to suppress the interference of the auxiliary sensors with low signal quality on the positioning algorithm. This method optimizes the proportion of the different sensor signals in the positioning algorithm with the BWF, thereby improving the accuracy of impact positioning. On the 300 mm × 300 mm × 15 mm sandwich panel specimen, the positioning accuracy reached 96.7% and the average positioning error was 0.85 mm. The present research provides a feasible method for impact positioning for carbon-fiber honeycomb panels with aluminum honeycombs.

The results of the experiment are as follows:

1. The division of the measurement area and the introduction of BWF can improve the performance of the localization method.
2. Original DPL, FDDL and ELM algorithms have achieved decent results but the result of SVM is not satisfactory. The results of B-DPL and B-FDDL are better than that of the above methods.
3. The increase in training samples can improve the classification performance of all the algorithms in the experiment. Among them, B-DPL and B-FDDL have the least dependence on the number of training samples.
4. Compared with B-FDDL, B-DPL has a faster calculation speed and is more suitable for impact positioning scenarios.

In the proposed method, the proportion of different sensors can be adjusted not only in the impact positioning, but also in other measurement scenarios if the signal qualities measured by different sensors are uneven. The idea of optimizing sensor configuration and proportion by dividing the measurement area and design sub-classifiers could be promoted in those measurement scenarios. 

This method compensates for the insufficiency of the time–domain performance of the measurement system by increasing the number of samples collected, which leads to a large amount of data-collection work during the training phase, and this is a shortcoming of the method. Furthermore, this method does not optimize the layout of the sensor network, and the division of the measurement area is relatively simple. Therefore, optimizing the sensor layout and measurement-area division will be the direction of the next stage of research.

## Figures and Tables

**Table 1:** Fiber Bragg grating (FBG) series of connections, wavelengths, and paste positions.

FBG No.	Initial Center Wavelength *λ* [nm]	Position [mm]
FBG1	1529.823	(−72, 72)
FBG2	1560.032	(0, 72)
FBG4	1535.023	(−72, 0)
FBG7	1549.933	(−72, −72)
FBG10	1544.421	Temperature compensation
FBG3	1524.961	(72, 72)
FBG5	1560.097	(0, 0)
FBG6	1529.788	(72, 0)
FBG8	1555.114	(0, −72)
FBG9	1549.760	(72, −72)

**Table 2:** Sub-areas and sensor groupings.

Sub-Area	Main Sensors	Auxiliary Sensors
1	FBG2, FBG3, FBG5, FBG6	FBG1, FBG4, FBG7, FBG8, FBG9
2	FBG3, FBG5, FBG6, FBG9	FBG1, FBG2, FBG4, FBG7, FBG8
3	FBG5, FBG6, FBG8, FBG9	FBG1, FBG2, FBG3, FBG4, FBG7
4	FBG5, FBG7, FBG8, FBG9	FBG1, FBG2, FBG3, FBG4, FBG6
5	FBG4, FBG5, FBG7, FBG8	FBG1, FBG2, FBG3, FBG6, FBG9
6	FBG1, FBG4, FBG5, FBG7	FBG2, FBG3, FBG6, FBG8, FBG9
7	FBG1, FBG2, FBG4, FBG5	FBG3, FBG6, FBG7, FBG8, FBG9
8	FBG1, FBG2, FBG3, FBG5	FBG4, FBG6, FBG7, FBG8, FBG9

**Table 3:** The length of principal component analysis (PCA) features and feature ratio in eight sub-areas.

Sub-Area	Features of Main Sensors	Feature Ratio%	Features of Auxiliary Sensors	Feature Ratio%
1	42/1024	90.29	37/1280	90.42
2	45/1024	90.43	39/1280	90.08
3	41/1024	90.88	38/1280	90.77
4	42/1024	90.31	40/1280	90.36
5	39/1024	90.27	39/1280	90.51
6	40/1024	90.13	34/1280	90.34
7	44/1024	90.15	40/1280	90.31
8	41/1024	90.42	38/1280	90.02

**Table 4:** Balancing weight factor (BWF) for each sub-area.

Sub-Area	1	2	3	4	5	6	7	8
BWF	0.35	0.25	0.35	0.30	0.30	0.40	0.35	0.45

## Data Availability

Not applicable.

## Nomenclature

The list of nomenclatures and symbols in this article

$\;\lambda_{B}$	Bragg wavelength of FBG
$\;\Delta \lambda_{B}$	Shifting of Bragg wavelength
n	Average refractive index of the fiber
$\;P_{ij}$	Pockel coefficients of the stress-optic tensor
v	Poisson’s ratio of the fiber
α	Thermal expansion coefficient of the fiber
ε	Strain on the sensor
ΔT	Change in temperature
$\;P_{e}$	Elasto-optic coefficient
λ	Initial center wavelength
Y	Training sample
$Y_{i}$	Training sample of each category
y	Test sample
D	Synthesis dictionary
$D_{i}$	Sub synthesis dictionary
$D^{*}$	Learned synthesis dictionary
X	Coding coefficient matrix
$X^{*}$	Learned coding coefficient matrix
P	Learned analysis dictionary
$P_{i}$	Sub analysis dictionary
$P^{*}$	Learned analysis dictionary
$\Psi \left(D,P,X\right)$	Constraint item related to the discriminative ability
*k*	Number of categories
m	Number of atoms in each category
γ	Adjusts weight
τ	Scalar constant of reconstruction error
$w_{B}$	Balancing weight factor
$W_{B}$	Balancing weight vector
